# Development and validation of routine clinical laboratory data derived marker-based nomograms for the prediction of 5-year graft survival in kidney transplant recipients

**DOI:** 10.18632/aging.202748

**Published:** 2021-03-26

**Authors:** Yamei Li, Lin Yan, Yi Li, Zhengli Wan, Yangjuan Bai, Xianding Wang, Shumeng Hu, Xiaojuan Wu, Cuili Yang, Jiwen Fan, Huan Xu, Lanlan Wang, Yunying Shi

**Affiliations:** 1 Department of Laboratory Medicine/Research Centre of Clinical Laboratory Medicine, West China Hospital, Sichuan University, Chengdu, China; 2 Department of Urology/Organ Transplant Center, West China Hospital, Sichuan University, Chengdu, China; 3 Department of Nephrology, West China Hospital, Sichuan University, Chengdu, China

**Keywords:** kidney transplantation, 5-year renal graft survival, prediction, nomogram, routine laboratory test data

## Abstract

Background: To develop and validate predictive nomograms for 5-year graft survival in kidney transplant recipients (KTRs) with easily-available laboratory data derived markers and clinical variables within the first year post-transplant.

Methods: The clinical and routine laboratory data from within the first year post-transplant of 1289 KTRs was collected to generate candidate predictors. Univariate and multivariate Cox analyses and LASSO were conducted to select final predictors. X-tile analysis was applied to identify optimal cutoff values to transform potential continuous factors into category variables and stratify patients. C-index, calibration curve, dynamic time-dependent AUC, decision curve analysis, and Kaplan-Meier curves were used to evaluate models’ predictive accuracy and clinical utility.

Results: Two predictive nomograms were constructed by using 0–6- and 0–12- month laboratory data, and showed good predictive performance with C-indexes of 0.78 and 0.85, respectively, in the training cohort. Calibration curves showed that the prediction probabilities of 5-year graft survival were in concordance with actual observations. Additionally, KTRs could be successfully stratified into three risk groups by nomograms.

Conclusions: These predictive nomograms combining demographic and 0–6- or 0–12- month markers derived from post-transplant laboratory data could serve as useful tools for early identification of 5-year graft survival probability in individual KTRs.

## INTRODUCTION

Benefiting from novel immunosuppressive agents and improved post-transplant management, kidney transplant recipients (KTRs) achieved excellent short-term survival, with a 1-year graft survival rate exceeding 95% [1]. Unfortunately, long-term graft survival has not shown synchronous improvement and remains a critical challenge for both patients and clinicians [2]. Among a variety of factors that contribute to such undesirable outcomes, the lack of a robust prediction system for late renal allograft loss is an important issue that needs to be addressed first. Without reliable evidence of risk stratification, clinicians cannot carry out efficient medical intervention in advance (such as modifying follow-up strategies and adjusting immunosuppression regimens) to prevent the upcoming deterioration of allograft function. In addition, prospective clinical trials on novel preventive or therapeutic agents might be limited due to the absence of early surrogate endpoints that can precisely estimate the risk of late graft failure in KTRs. Therefore, accurately predicting and identifying the probability of allograft loss preemptively is essential to guiding individualized clinical therapeutic decisions in KTRs.

Although a relatively large number of prognostic models have been developed and validated to predict graft failure in KTRs [3, 4], few have been widely implemented in clinical settings due to certain limitations. For example, some promising models were developed and validated on the basis of populations from Occident medical centers [5–7], where patients’ genetic backgrounds, transplant-related management strategies, and health insurance are different from those in Eastern countries. Such a gap may affect their general applicability among transplant centers around the world. Further rigorous validation is necessary or, as an alternative, new prognostic models should be constructed on the basis of local patients to facilitate native clinical application. In addition, many models only include static predictors measured at transplantation, such as recipient and donor demographics and transplant-related parameters [8, 9], or from later on during follow-up, for instance, renal function markers (serum creatinine and urine protein) at a single referring post-transplant point [10–12]. However, limited studies utilized omnibus longitudinal data as predictors [6] despite the fact that this data is the main source of clinical decision-making. Recently, some notable findings demonstrated that routine laboratory index-derived markers that reflect the overall state of a certain period time could serve as valuable predictors for poor outcomes in KTRs. Higher tacrolimus (TAC) trough concentration derived markers, TAC-intrapatient variability (TAC-IPV or TAC-CV) [13, 14], and TAC-time in therapeutic range (TAC-TTR) [15], were extensively proven to be strongly linked with subsequent composite poor outcomes in transplant patients. Furthermore, estimated glomerular filtration rate (eGFR) related novel markers including eGFR-coefficient of variation (eGFR-CV) [16, 17], as well as inflammatory markers calculated from complete blood count (CBC) data, such as neutrophil-to-lymphocyte ratio (NLR), platelet-to-lymphocyte ratio (PLR), and monocyte-to lymphocyte ratio (MLR) [18–20], were shown to be associated with adverse events in KTRs. Therefore, we hypothesized that adding these relatively new markers to prognostic models may provide additional information to improve the risk assessment of allograft loss in KTRs.

Taking into account the excellent 1-year graft survival rate and the ubiquitous follow-up strategy where CBC, urine routine test, renal function, and TAC trough concentration were regularly monitored in a majority of KTRs, we adopted the above easily obtained laboratory test data from within the first 6 or 12 months post-transplantation to generate new candidate predictors. The objective of our study was to develop and validate two prognostic nomogram models by combining laboratory data-derived risk factors with demographic and clinical variables for individually predicting the probability of 5-year kidney allograft survival in KTRs. In addition, to increase the practicability of these models, web servers and risk stratification systems were further established.

## RESULTS

### Characteristics of patients

**Figure 1:**
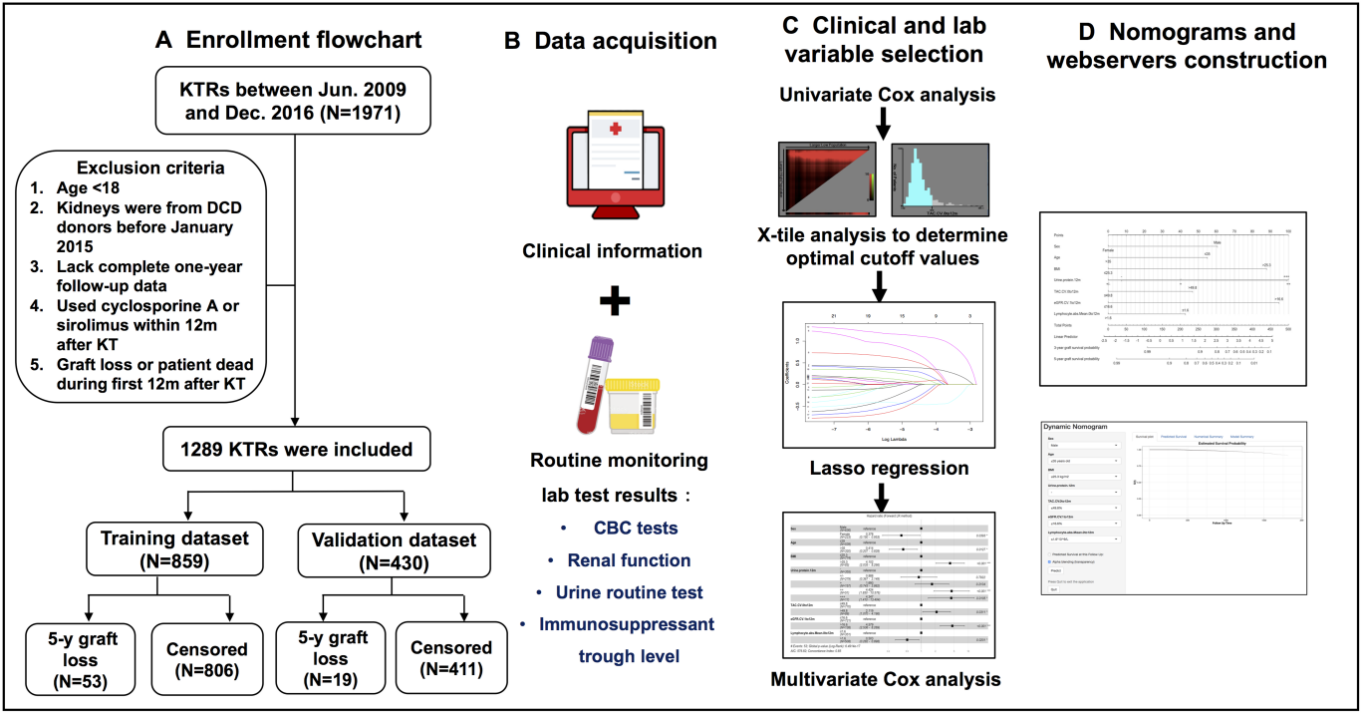
**Flow diagram of developing predictive nomograms for 5-year graft survival in KTRs.** (**A**) Patients enrollment flowchart. (**B**) Candidate predictor and outcome data acquisition. (**C**) Predictor selection process. (**D**) Nomogram and web server construction.

Of the initial 1971 KTRs, 1289 patients were included in the final study after multiple rounds of exclusion according to the established criteria (Figure 1A). Among the selected patients, 859 KTRs, with 53 KTRs suffering from 5-year graft loss, were randomly assigned to the training cohort, while the remaining 430 KTRs, with 19 KTRs having confirmed 5-year graft loss, were included in the validation cohort. The median follow-up date for censored KTRs was 1239 days in the training cohort and 1269 days in the validation cohort. The time point distribution of occurring graft loss in training and validation cohorts is displayed in Supplementary Figure 2. Additionally, 116 KTRs in the training cohort and 52 KTRs in the validation cohort lacked height data, so multiple imputation methods incorporating gender, age, race, and outcomes were applied to impute appropriate values, and BMI was thereby generated for the following analysis. Table 1 presents the statistical comparison of patient demographics and laboratory variables between training and validation cohorts. In brief, the training cohort comprised a higher number of minorities and showed higher average levels of anemia-related indicators (RBC, HGB, and HCT) within the first 6 or 12 months when compared to those in the validation cohort. No differences were observed regarding other variables.

**Table 1.** Baseline clinical characteristics and laboratory indexes of patients in training and validation cohorts.

**Variables**	**Training set (*N* = 859)**	**Validation set (*N* = 430)**	***P* value**
***Clinical variables***			
Sex (M/F)	636/223	316/114	0.840
Age (years)	32 (26–39)	31 (26–41)	0.472
Race (Han/Tibetan/Others)	757/54/48	389 /33/8	**0.006**
BMI (kg/m^2^)	20.76 (18.90–23.08)	20.41 (18.73–22.49)	0.091
Pre-transplant Urine output (<100/100–400/400–1000/>1000 ml)	327/255/184/93	160/129/93/48	0.992
Dialysis type (None/Hemodialysis/Peritoneal dialysis)	62/734/63	32/370/28	0.858
Dialysis duration (months)	11 (6–21)	11 (6–21)	0.577
Donor type (Living-related/Living-unrelated/Deceased)	700/65/94	353/30/47	0.929
HLA mismatch (A, B, DR, DQ) (0–2/3–5/6–8)	126/602/131	52/310/68	0.450
Hospitalization time (days)	25 (22–30)	24 (22–31)	0.883
DGF (Y/N)	827/32	409/21	0.372
Acute rejection (0 to 6m) (Y/N)	11/848	10/420	0.168
Acute rejection (0 to 12m) (Y/N)	21/838	12/418	0.711
Hospitalization number due to infection (0 to 6m) (Y/N)	95/764	35/395	0.116
Hospitalization number due to infection (0 to 12m) (Y/N)	115/744	44/386	0.107
***Laboratory indexes within 6 months***			
Urine protein at month 6			0.226
–	390	217	
–/+	286	144	
+	153	57	
++	22	9	
+++	8	3	
TAC-Mean (0 to 6m) (ng/mL)	6.27 (5.32–6.93)	6.19 (5.25–6.87)	0.551
TAC-CV (0 to 6m) (%)	31.99 (26.54–39.75)	33.73 (27.53–41.30)	0.060
TAC-loLL (0 to 6m) (%)	23.08 (11.76–39.45)	23.31 (12.50–41.18)	0.328
TAC-TTR (0 to 6m) (%)	66.67 (53.33–78.57)	64.71 (50.00–76.92)	0.162
TAC-hiUL (0 to 6m) (%)	6.25 (0.00–13.33)	5.88 (0.00–13.33)	0.923
eGFR-Mean (1 to 6m) (mL/min/1.73m^2^)	75.57 (64.26–86.56)	73.91 (63.65–86.44)	0.429
eGFR-CV (1 to 6m) (%)	10.30 (8.17–13.53)	10.50 (8.30–13.68)	0.346
Uric acid-Mean (0 to 6m) (umol/L)	327.67 (288.66–364.45)	326.77 (286.14–375.83)	0.647
RBC-Mean (0 to 6m) (10^12/L)	4.48 (4.09–4.89)	4.38 (4.06–4.80)	**0.030**
HGB-Mean (0 to 6m) (g/L)	131.35 (120.00–142.75)	128.50 (119.50–140.40)	**0.037**
HCT-Mean (0 to 6m) (L/L)	0.41 (0.38–0.44)	0.40 (0.38–0.44)	**0.036**
PLT-Mean (0 to 6m) (10^9/L)	179.18 (137.00–225.22)	178.82 (143.38–216.00)	0.868
WBC-Mean (0 to 6m) (10^9/L)	6.99 (5.98–8.14)	6.98 (5.85–8.33)	0.932
Neutrophil-Mean (0 to 6m) (%)	64.52 (59.41–69.08)	64.48 (59.83–69.00)	0.976
Lymphocyte-Mean (0 to 6m) (%)	25.36 (21.37–30.68)	25.53 (21.35–30.75)	0.788
Monocyte-Mean (0 to 6m) (%)	7.33 (6.33–8.46)	7.21 (6.37–8.33)	0.461
Neutrophil-Mean (0 to 6m) (10^9/L)	4.52 (3.65–5.37)	4.39 (3.67–5.43)	0.545
Lymphocyte-Mean (0 to 6m) (10^9/L)	1.77 (1.37–2.19)	1.81 (1.38–2.19)	0.795
Monocyte-Mean (0 to 6m) (10^9/L)	0.50 (0.41–0.59)	0.50 (0.41–0.59)	0.625
NLR-Mean (0 to 6m)	2.75 (2.09–3.64)	2.75 (2.05–3.71)	0.967
PLR-Mean (0 to 6m)	107.77 (79.12–148.30)	109.11 (79.56–147.51)	0.962
MLR-Mean (0 to 6m) (%)	30.17 (23.64–38.77)	30.08 (22.98–40.20)	0.940
***Laboratory indexes within 12 months***			
Urine protein at month 12			0.647
–	355	185	
–/+	279	149	
+	157	68	
++	51	22	
+++	17	6	
TAC-Mean (0 to 12m) (ng/mL)	6.10 (5.34–6.67)	6.07 (5.24–6.70)	0.569
TAC-CV (0 to 12m) (%)	32.89 (27.98–39.70)	33.68 (27.92–40.23)	0.431
TAC-loLL (0 to 12m) (%)	20.69 (12.06–35.00)	21.98 (11.54–36.84)	0.513
TAC-TTR (0 to 12m) (%)	68.75 (57.14–78.26)	67.76 (54.84–77.42)	0.227
TAC-hiUL (0 to 12m) (%)	6.67 (0.00–13.04)	6.90 (2.50–13.33)	0.908
eGFR-Mean (1 to 12m) (mL/min/1.73m^2^)	75.57 (64.88–86.85)	74.07 (63.74–86.29)	0.337
eGFR-CV (1 to 12m) (%)	11.09 (9.03–14.05)	11.26 (9.18–14.21)	0.564
Uric acid-Mean (0 to 12m) (umol/L)	340.96 (300.35–380.86)	340.96 (296.21–387.85)	0.834
RBC-Mean (0 to 12m) (10^12/L)	4.62 (4.22–5.07)	4.50 (4.17–4.96)	**0.021**
HGB-Mean (0 to 12m) (g/L)	135.06 (124.60–147.13)	132.26 (122.59–144.75)	**0.022**
HCT-Mean (0 to 12m) (L/L)	0.42 (0.39–0.46)	0.42 (0.39–0.45)	**0.015**
PLT-Mean (0 to 12m) (10^9/L)	175.95 (136.58–221.60)	179.14 (141.91–215.00)	0.921
WBC-Mean (0 to 12m) (10^9/L)	7.07 (6.01–8.16)	7.03 (6.03–8.17)	0.958
Neutrophil-Mean (0 to 12m) (%)	65.02 (60.07–69.17)	64.63 (60.17–69.14)	0.740
Lymphocyte-Mean (0 to 12m) (%)	25.31 (21.28–30.38)	25.57 (21.35–30.29)	0.538
Monocyte-Mean (0 to 12m) (%)	7.37 (6.43–8.40)	7.26 (6.38–8.30)	0.468
Neutrophil-Mean (0 to 12m) (10^9/L)	4.59 (3.72–5.39)	4.46 (3.76–5.36)	0.547
Lymphocyte-Mean (0 to 12m) (10^9/L)	1.76 (1.39–2.18)	1.80 (1.39–2.15)	0.762
Monocyte-Mean (0 to 12m) (10^9/L)	0.50 (0.42–0.60)	0.50 (0.43–0.59)	0.551
NLR-Mean (0 to 12m)	2.79 (2.17–3.71)	2.79 (2.16–3.70)	0.950
PLR-Mean (0 to 12m)	106.71 (80.86–147.48)	107.53 (80.20–144.02)	0.906
MLR-Mean (0 to 12m) (%)	30.42 (24.48–39.08)	30.68 (23.51–40.12)	0.897

Abbreviations: BMI: body mass index; DGF: delayed graft function; HLA: human leukocyte antigen; TAC: tacrolimus; CV: coefficient of variation; loLL: lower than lower limit; TTR: time in therapeutic range; hiUL: higher than upper limit; RBC: red blood cell; HGB: hemoglobin; HCT: hematocrit; PLT: platelet; WBC: white blood cell; NLR; neutrophil to lymphocyte ratio; PLR: platelet to lymphocyte ratio; MLR: monocyte to lymphocyte ratio.

### Feature selection

**Figure 2:**
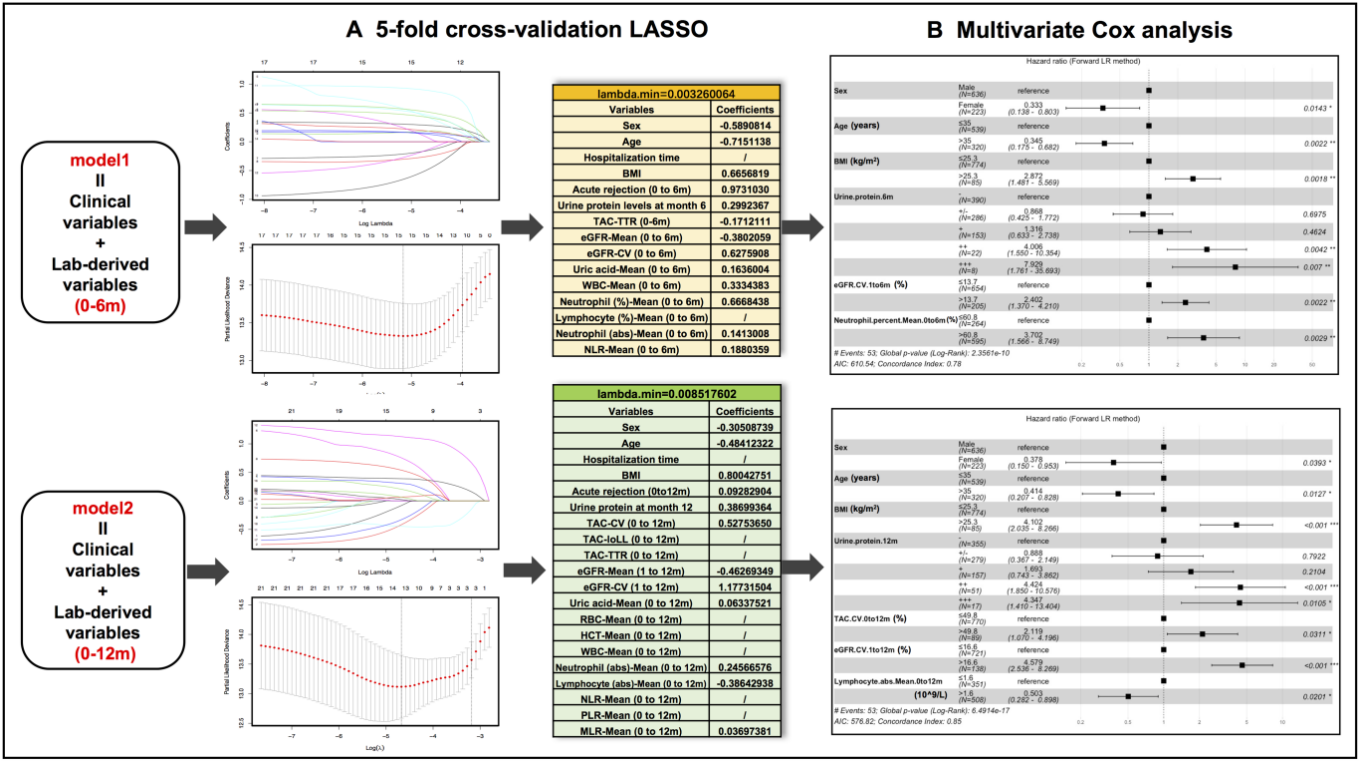
**Predictive variable selection results in model 1 (clinical variables + months 0-6 lab-derived variables) and model 2 (clinical variables + months 0-12 lab-derived variables) by LASSO regression and multivariate Cox regression analyses.** (**A**) Preliminary selected variables in model 1 and model 2 by 5-fold cross-validation LASSO analysis. (**B**) Hazard ratio forest plots of final predictors in model 1 and model 2.

The associations between candidate predictors consisting of 13 demographic and clinical variables and 46 laboratory data-derived variables (23 calculated with 0–6m data and 23 with 0–12m data)] and outcomes were preliminarily screened in univariate Cox regression analysis (Table 2). Of the 59 variables, 31 variables showed *P* value < 0.15 and were stratified based on optimal cut-off values obtained from X-tile analysis (Supplementary Figure 1). The stratified demographic and clinical variables (*n* = 6) combined with stratified 0–6m laboratory (*n* = 10) and 0–12m laboratory variables (*n* = 15) were grouped into model 1 and model 2, respectively. After LASSO regression analyses, 13 variables from model 1 and 12 variables from model 2 were separately entered into the multivariate Cox regression analysis (Figure 2A). Finally, 6 predictors (sex, age, BMI, urine protein level at month 6, eGFR-CV.1 to 6m, and neutrophil percent-Mean.0to6m) were selected in the final model 1 and 7 predictors (sex, age, BMI, urine protein level at month 12, TAC-CV.0 to 12m, eGFR-CV.1 to 12m, and lymphocyte absolute number-Mean.0 to 12m) were included in the final model 2 (Figure 2B).

**Table 2.** Univariate Cox hazards analysis of the training cohort.

**Variables**	**HR (95%CI)**		***P* value**	
***Clinical variables***				
Female	0.336 (0.144–0.786)		**0.012**	
Age (years)	0.964 (0.932–0.998)		**0.037**	
Race				
Han	1.000			
Tibetan	0.961 (0.299–3.09)		0.947	
Others	1.159 (0.360–3.729)		0.804	
BMI (kg/m^2^)	1.115 (1.031–1.206)		**0.006**	
Pre-transplant Urine output				
<100 ml	1.000			
100–400 ml	1.205 (0.652–2.225)		0.552	
400–1000 ml	0.450 (0.181–1.123)		**0.087**	
>1000 ml	0.733 (0.293–1.833)		0.506	
Dialysis type				
None	1.000			
Hemodialysis	1.781 (0.552–5.743)		0.334	
Peritoneal dialysis	1.952 (0.436–8.742)		0.382	
Dialysis duration (months)	0.993 (0.975–1.011)		0.431	
Donor type				
Living-related	1.000			
Living-unrelated (spouse)	1.105 (0.438–2.784)		0.833	
Deceased	1.116 (0.337–3.698)		0.857	
HLA mismatch (A, B, DR, DQ)				
0–2	1.000			
3–5	1.519 (0.647–3.569)		0.337	
6–8	0.789 (0.222–2.798)		0.713	
Hospitalization time (days)	1.026 (0.992–1.060)		**0.134**	
DGF	1.243 (0.302–5.114)		0.763	
Acute rejection (0 to 6m)	9.012 (2.763–29.396)		**<0.001**	
Acute rejection (0 to 12m)	9.012 (2.763–29.396)		**<0.001**	
Hospitalization number due to infection (0 to 6m)	1.149 (0.491–2.689)		0.748	
Hospitalization number due to infection (0 to 12m)	1.652 (0.830–3.289)		0.153	
**Variables**	**Laboratory indexes within 6 months**		**Laboratory indexes within 12 months**	
	**HR (95%CI)**	***P* value**	**HR (95%CI)**	***P* value**
Urine protein at month 6/12				
–	1.000		1.000	
–/+	1.080 (0.537–2.172)	0.829	0.953 (0.402–2.263)	0.914
+	1.947 (0.954–3.974)	**0.067**	2.703 (1.250–5.844)	**0.012**
++	7.287 (2.888–18.390)	**<0.001**	8.577 (3.912–18.805)	**<0.001**
+++	8.967 (2.072–38.812)	**0.003**	9.496 (3.337–27.019)	**<0.001**
TAC-Mean (ng/mL)	0.975 (0.777–1.224)	0.831	0.893 (0.687–1.160)	0.395
TAC-CV (%)	1.011 (0.992–1.030)	0.244	1.023 (1.005–1.040)	**0.012**
TAC-loLL (%)	1.006 (0.994–1.019)	0.335	1.014 (0.999–1.028)	**0.064**
TAC-TTR (%)	0.988 (0.975–1.002)	**0.089**	0.982 (0.967–0.998)	**0.025**
TAC-hiUL (%)	1.017 (0.991–1.044)	0.208	1.006 (0.977–1.036)	0.701
eGFR-Mean^*^ (mL/min/1.73m^2^)	0.978 (0.961–0.995)	**0.013**	0.957 (0.939–0.975)	**<0.001**
eGFR-CV^*^ (%)	1.043 (1.013–1.073)	**0.004**	1.078 (1.057–1.100)	**<0.001**
Uric acid-Mean (umol/L)	1.004 (1.000–1.008)	**0.073**	1.006 (1.002–1.011)	**0.007**
RBC-Mean (10^12/L)	1.059 (0.674–1.662)	0.804	0.697 (0.447–1.084)	**0.109**
HGB-Mean (g/L)	1.005 (0.989–1.022)	0.508	0.990 (0.975–1.005)	0.197
HCT-Mean (L/L)	1.490 (0.006–361.852)	0.887	0.007 (0.000–1.238)	**0.060**
PLT-Mean (10^9/L)	1.002 (0.998–1.006)	0.309	1.002 (0.998–1.007)	0.252
WBC-Mean (10^9/L)	1.128 (0.974–1.306)	**0.107**	1.171 (1.008–1.361)	**0.038**
Neutrophil-Mean (%)	1.034 (0.998–1.071)	**0.067**	1.000 (0.999–1.001)	0.677
Lymphocyte-Mean (%)	0.965 (0.930–1.001)	**0.059**	1.000 (0.999–1.001)	0.685
Monocyte-Mean (%)	0.867 (0.713–1.054)	0.152	1.000 (0.999–1.001)	0.755
Neutrophil-Mean (10^9/L)	1.216 (1.018–1.451)	**0.031**	1.338 (1.127–1.588)	**0.001**
Lymphocyte-Mean (10^9/L)	0.904 (0.632–1.292)	0.579	0.732 (0.479–1.118)	**0.149**
Monocyte-Mean (10^9/L)	1.791 (0.255–12.583)	0.558	1.883 (0.259–13.693)	0.532
NLR-Mean	1.083 (0.978–1.198)	**0.126**	1.192 (1.103–1.289)	**<0.001**
PLR-Mean	1.002 (0.999–1.005)	0.295	1.004 (1.001–1.008)	**0.017**
MLR-Mean (%)	1.007 (0.998–1.027)	0.448	1.020 (1.001–1.039)	**0.035**

Note: ^*^eGFR-Mean and –CV values were calculated based on month 1 to 6 eGFR data, other variables were based on month 0 to 6 data.

### Nomogram construction, assessment and validation

**Figure 3:**
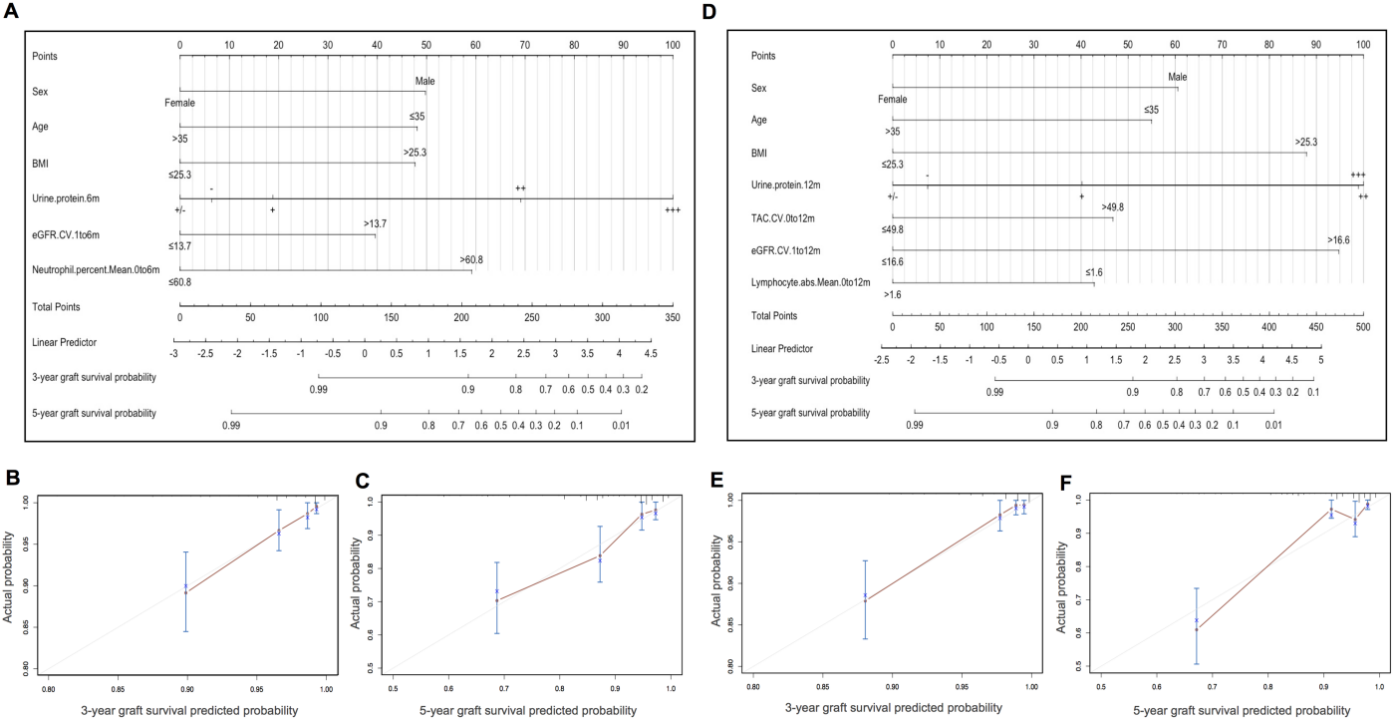
**Nomograms and calibration curves of model 1 (clinical variables + months 0-6 lab-derived variables) and model 2 (clinical variables + months 0-12 lab-derived variables) for predicting 3- and 5-year graft survival in the training cohort.** (**A**) Nomogram for model 1. (**B–C**). Calibration curves of the model 1 nomogram for 3- and 5-year graft survival. (**D**) Nomogram for model 2. (**E–F**) Calibration curves of the model 2 nomogram for 3- and 5-year graft survival.

The results of prognostic model 1 and model 2 were visually mapped by nomograms to predict 3- and 5-year graft survival of KTRs (Figure 3A and 3D). To make these models easier to use, laboratory predictors can be calculated by simply inputting original test results into a web calculator available via https://docs.google.com/spreadsheets/d/1IJX9YZBTON1xwVrNYp5PzcNpWQ1CItGm-N_nDOKxbpk/edit?pli=1#gid=0. Web servers of nomograms can be accessed through https://yameili.shinyapps.io/model1for5yeargraftsurvival/ for model 1 and https://yameili.shinyapps.io/ model2for5yeragraftsurvival/ for model 2. Calibration plots of two nomograms for the probability of 3- and 5-year graft survival showed good agreement between actual observations and nomogram predictions (Figure 3B, 3C and 3E, 3F).

**Figure 4:**
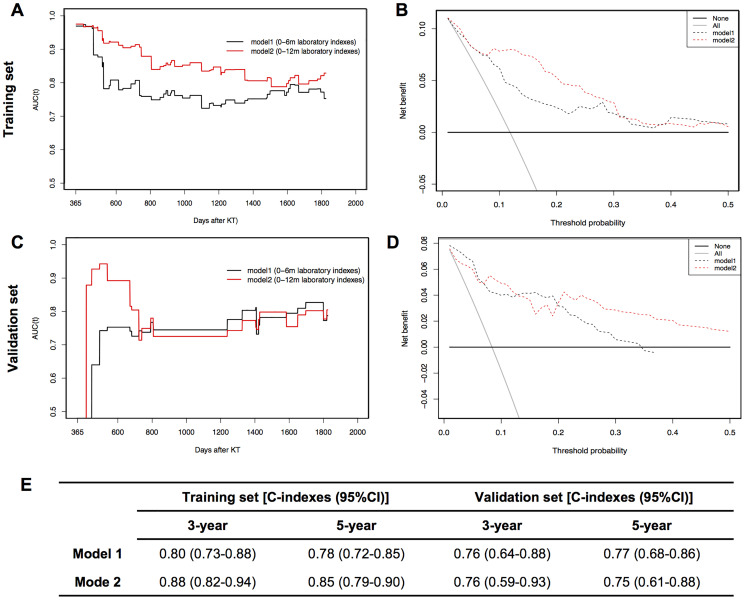
**Dynamic time-dependent AUC (TD-AUC) curves, decision curve analysis (DCA) and summary of C-indexes of two models in training and validation sets.** (**A**). TD-AUC curves in the training set. (**B**) DCA curves in the training set. (**C**). TD-AUC curves in the validation set. (**D**) DCA curves in the validation set. (**E**) C-indexes summary of model 1 and model 2 for predicting 3- and 5-year graft survival in training and validation sets.

As summarized in Figure 4E, model 1 and model 2 nomograms achieved good performances in predicting 3- and 5-year graft survival as evidenced by C-indexes higher than 0.75 in both training and validation cohorts. In addition, model 2 showed better discriminative power than model 1 in the training set, but failed to be verified in the validation set. TD-AUC curves were performed to dynamically present the predictive abilities of nomograms at serial time points. All AUC values of model 1 (Figure 4A) and model 2 (Figure 4C) were higher than 0.7 during the observation period in both the training and validation sets, showing good predictive power in predicting graft loss. Moreover, model 2 outperformed model 1 in the training cohort but showed similar predictive capacity to model 1 in the validation cohort. According to DCA plots (Figure 4B and 4D), when the threshold probability for a patient was within 0.0 to 0.5, model 1 and model 2 nomograms showed more net benefit than “treat all” or “treat none” strategies, indicating that they were feasible to make valuable and profitable clinical judgments.

### Performance of the nomogram in stratifying risk of KTRs

**Figure 5:**
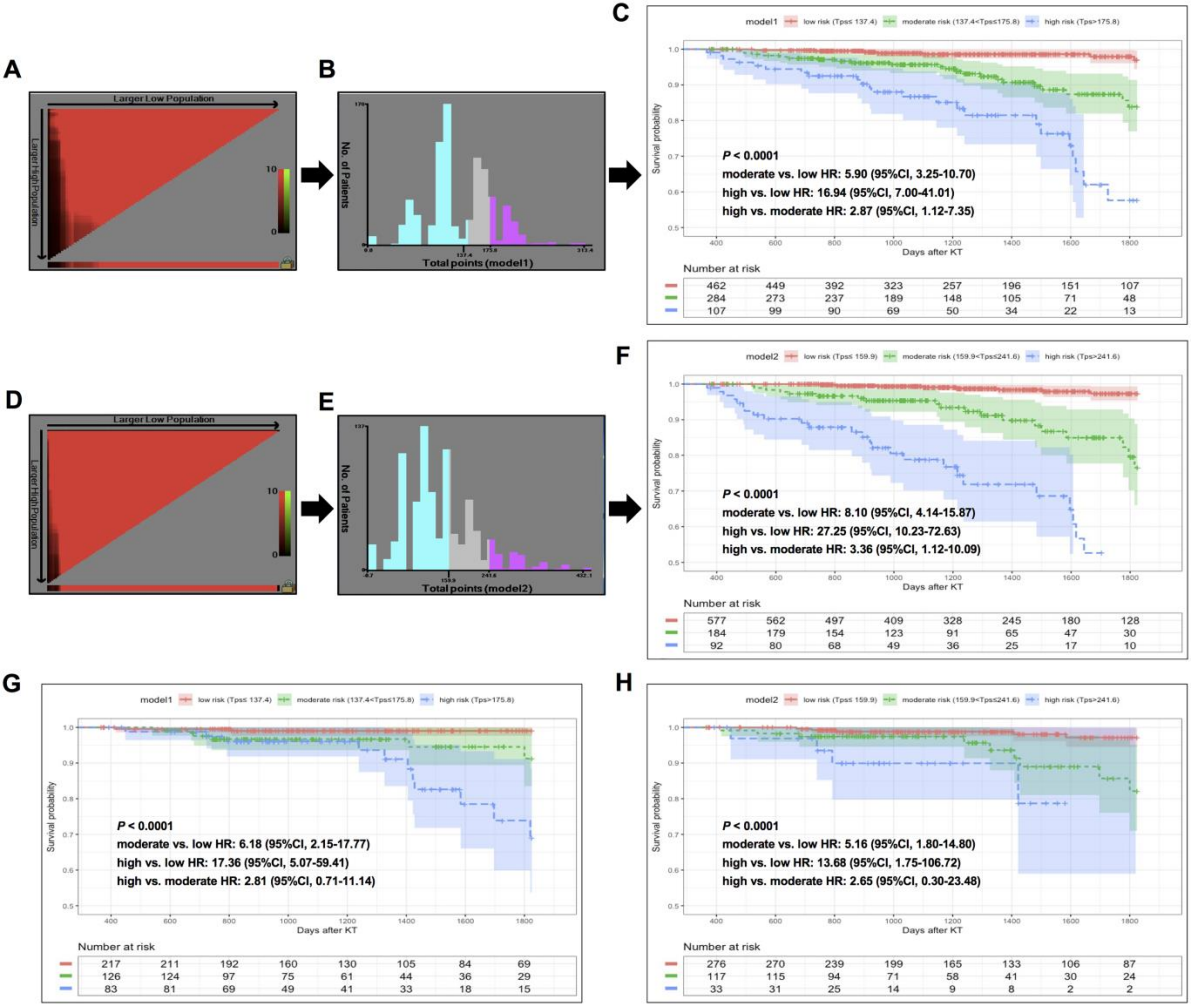
**X-tile analysis of total risk score and survival curves stratified by the score calculated from nomogram scoring systems in training and validation sets.** In training set: (**A–B**) X-tile plot and total points histogram showing the optimal cut-points of model 1. (**C**) Kaplan-Meier (KM) survival curve of different risk groups based on cut-points obtained from model 1 X-tile analysis. (**D–E**) X-tile plot and total points histogram showing the optimal cut-points of model 2. (**F**) KM survival curve of different risk groups based on cut-points obtained from model 2 X-tile analysis. In validation set: (**G–H**). KM survival curves of KTRs stratified by optimal cut-points from model 1 (G) and model 2 (H).

In the training cohort, patients were classified into three risk groups based on total risk scores calculated by model 1 (low-risk, ≤137.4; moderate-risk, 137.4~175.8; high-risk, >175.8) and model 2 (low-risk, ≤159.9; moderate-risk, 159.9~241.6; high-risk, >241.6) nomograms following the determination of cutoff values by X-tile analyses (Figure 5A, 5B; 5D, 5E). Compared to low-risk populations stratified by the model 1 system, HRs (95% CI) for moderate- and high-risk KTRs were 5.90 (3.25–10.70) and 16.94 (7.00–41.01) in the training cohort (Figure 5C), and 6.18 (2.15–17.77) and 17.36 (5.07–59.41) in the validation cohort (Figure 5G). When stratified by the model 2 system, HRs (95%CI) for moderate- and high-risk KTRs were 8.10 (4.14–15.87) and 27.25 (10.23–72.63) in the training cohort (Figure 5F), and 5.16 (1.80–14.80) and 13.68 (1.75–106.72) in the validation cohort (Figure 5H). The Kaplan-Meier curves indicated that both nomograms achieved successful risk stratification in KTRs with statistical significance (*P* < 0.0001).

## DISCUSSION

In this study, we successfully developed and validated two novel nomogram-based prognostic models that were capable of individually predicting 5-year allograft loss in KTRs. These nomograms combined early, routinely accessible, yet significant laboratory test-derived indicators (calculated based on 0–6m and 0–12m laboratory results) and demographic variables to generate accurate prognostic predictions of individual patients. Such predictions presented a good ability to discriminate KTRs with low-, moderate-, and high-risk of developing forthcoming graft loss within 1 to 5 years post-transplant.

In the last decade, a growing body of prognostic scoring systems have been reported for the early prediction of long-term renal allograft survival on the basis of various predictors, the majority of which were “static” demographical and clinical variables at certain time points before or after transplantation. Such static Cox or Logistic models incorporating variables measured at time 0 to predict the events occurred throughout the full evaluation period, which might be less effective than using longitudinal data in predicting future risk [21]. More recently, an internationally derived and robustly validated (in Europe and North America centers) prediction system for estimating the risk of graft loss in KTRs was constructed by combining 8 functional, histological and immunological prognostic factors. Strong predictive ability has been shown with C-indexes exceeding 0.8 in different clinical scenarios and subpopulations [7]. However, considering distinct patients’ characteristics, clinical management practices, and the unavailability of histological data for the majority of KTRs in most Chinese transplant centers, it may not be feasible for the implementation in our routine clinical settings. Taking these issues into account, we turned our attention to “dynamic” risk factors which were derived from serial routinely monitored laboratory examination results. We applied multiple calculation forms including average, CV, and proportions of time within or out of therapeutic range to process original laboratory data during early periods post-transplantation. By combining demographic and clinical risk factors with 0-6m and 0-12m processed laboratory predictors, we built up model 1 and model 2 prognostic nomograms, respectively. Both models showed moderate to strong predictive efficacy for early identification of 5-year graft loss, and model 2 was superior to model 1 (C-index for model 1 was 0.78 (0.72–0.85) and for model 2 was 0.85 (0.79–0.90)). We did not conduct direct comparisons between the present study and previously described risk scoring systems due to the lack of some predictor information, such as blood pressure data in DuBay DA’s study [6]. However, when only considering data from a C-index and TD-AUC perspective, our models showed higher values. Taken together, these results indicated that comprehensively integrating laboratory variables was a novel strategy to find risk predictors.

In our two prognostic nomograms, recipient characteristics including female gender, older age, and lower BMI levels were independent protective factors of long-term graft survival. However, there were some controversies regarding the associations between those individual predictors and renal allograft outcomes. The findings on being female were consistent with some of previous studies [6, 22, 23], but differed from Yohann Foucher [5] and H.Y.Tiong’s studies [24], in which being female was closely associated with poor allograft outcomes in KTRs. Such inconsistency also exists in recipient age and BMI variables among this and other different KTR cohorts [5, 22]. These discordant results suggest that constructing personalized nomograms that host “one size fits all” properties is not an easy task, which may be hampered by the heterogeneity of patient backgrounds and post-transplant management strategies.

In addition to those fixed baseline variables, some potentially amendable post-transplant risk factors were identified in this study. eGFR variability, no matter calculated from 1–6 months eGFR values or 1–12 months eGFR values, was a significant risk factor for graft loss and independent of average eGFR levels. In accordance with Nicole A. Pilch’s [16] and Hoon Young Choi et al’s. [17] studies, our results suggested that converting serial longitudinal eGFR data into eGFR-CV provided more prognostic information than just simply averaging them. Several possible factors may explain its superior ability in early presentations of signs of graft loss. eGFR variability is a rational marker of renal allograft homeostasis. Intrinsic renal factors (such as acute allograft rejection, subclinical rejection, and other damages that directly target the kidney) and extrinsic factors (such as infection, extra-renal organ system injury, and comorbid complications that indirectly affect renal function) may disturb the steady state of the kidney [25, 26]. Even though some deteriorative renal function may revert to its prior “normal” level after appropriate intervention, eGFR variability can still provide comprehensive information on both unmeasured and measurable detrimental events that may have side effects on subsequent graft survival. TAC intra-patient variability (TAC-IPV or TAC-CV) was another important risk factor for graft loss in our study. Accumulating studies have demonstrated that high TAC-CV was associated with poor allograft outcomes such as graft failure and chronic antibody-mediated rejection in various solid organ transplantations [13, 27–29]. Unlike previously published studies, and since we wanted to use TAC-CV to reflect the overall combined impact of medication non-adherence, food and drug interaction, genetic factors, and dose modification due to concurrent diseases or TAC-related toxicity throughout the entire interested post-transplant period [14], TAC-CV was calculated by adopting all TAC trough concentration data from within 0–6 or 0–12 months, rather than eliminating early-phase data that was thought to be unstable because of the frequent dose adjustment to achieve target levels during hospitalization. Final inclusion of TAC-CV in model 2, not in model 1, indicated that replenishing early TAC-CV with relatively late TAC concentration data would be conducive to improve its predictive ability for 5-year graft loss. Interestingly, we found a high average percentage of 0–6 months neutrophil and low 0–12 months lymphocyte mean count emerged as independent risk indicators for subsequent graft loss in different prediction models. Such abnormality of average peripheral leukocyte count may reflect the sustained excessive immunosuppression during certain time periods, which increases the risk of various infections and then threatens the long-term survival [30–32].

Several strengths could be found in the current study. First, our study took full advantage of easily accessible laboratory data during 6 or 12 months post-transplantation to generate novel and cost-effective potential predictors, some of which have been revealed to significantly correlate with poor long-term allograft survival in distinct population-based studies. Nomograms incorporating an early shorter or relatively longer period of laboratory indicators with demographic variables both demonstrated good discrimination and calibration power, which enabled the early identification of individual graft-loss risks without additional cost. Second, we set up the risk thresholds that can classify KTRs into low-, moderate-, and high- risk groups as early as 6 or 12 months after transplantation. This would be helpful to promote personalized health evaluations and carry out precise prevention by adjusting follow-up frequencies, adding protocol biopsies, or modifying immunosuppression regimens. In addition, it would also be favorable in conducting prospective clinical trials by allowing targeted recruitment of moderate- to high-risk patients. Third, considering the clinical practice, nomograms were further converted into web-based apps and predictor calculators were designed to facilitate clinical application.

However, several limitations should be addressed in this study. First, due to the nature of retrospective design, the small number of events, and the single center analysis, the results may be somewhat heterogeneous. Although we have validated these results in a random split-sample cohort, external validation studies from other institutions, or at least from other Chinese institutions, are definitely warranted to confirm these findings. Second, some features would potentially improve the predictive performance of models, such as warm and cold ischemia time and donor specific antibody levels, which were not included in this study due to the incomplete records in our database. Third, these models were only applicable for adult KTRs who maintained their graft function within the first year. However, for those who lost the grafts during the first year, pre- and peri-transplant, as well as early post-transplant parameters, would be feasible to achieve good predictive performance for this patient population.

In summary, we constructed and validated two novel nomograms by incorporating clinical variables and relatively new risk factors that derived from a serial of easily-available laboratory examination data from within the first half- or one-year post-transplant to identify the risk of 5-year graft loss in KTRs early. In addition, the nomogram-based classifiers could provide supportive information on stratifying individual patients into different risk groups, which may assist clinicians and patients in clinical decision-making and adjustment of post-transplant management strategies. Further external validation and prospective application of these models will be performed in our future studies.

## MATERIALS AND METHODS

### Participants

Patients who underwent kidney transplant surgery in West China Hospital between June 2009 and December 2016 were retrospectively included in this study. We chose this period because we could extract accurate electronic health record (EHR) data. KTRs who met any of the following criteria were excluded: (1) age at transplantation was less than 18 years old, (2) received kidneys from deceased donors before January 2015 since organ donation from voluntary civilian organ donors has become the only lawful source of organ transplantations, (3) had less than one-year follow-up or were without complete one-year laboratory test results, (4) cyclosporine A or sirolimus were used within the first 12 months after transplantation, (5) graft loss or patient death occurred in the first 12 months after transplantation. Eligible patients were then randomly split into training and validation datasets at a ratio of 2:1. All KTRs were treated with standard triple regimens (TAC + mycophenolate mofetil + steroid) as maintenance immunosuppression. Preventive anti-infective drugs were preoperatively and postoperatively administered, as previously described [33]. This study was approved by the institutional review board of West China Hospital [no.2017(397)]. Because of the retrospective nature of this study, informed consent from patients was waived.

### Endpoints and definitions

The primary outcome of this study was that allograft loss occurred between 1 year and 5 years post-transplantation. Outcome data was retrospectively retrieved from the hospital information system (HIS) on February 28, 2019. Graft loss events were defined as a return to maintenance dialysis, re-transplantation, uremia (eGFR<15 mL/min/1.73 m2 without recovery), or all-cause death. Time to event was recorded at the date of censoring (date of whichever graft loss event happened first or of the last follow-up date) or 1825 days (5 years) after transplantation. A clinical transplant nephrologist was invited to assess ambiguous outcomes.

### Candidate predictor acquisition and preprocessing

Patients’ demographic and clinical characteristics were collected: gender, age, race, body mass index (BMI), pre-transplant urine output, dialysis type, time on dialysis, donor type, human leukocyte antigen (HLA) mismatch, hospitalization duration after KT surgery, delayed graft function (DGF), acute rejection episode, and number of hospitalizations due to infection within the first 6 or 12 months post-transplantation.

One-year post-transplant original laboratory data including CBC test, renal function indicators, routine urine test, and immunosuppressant trough levels were extracted from the laboratory information system (LIS). This data was then processed to generate new candidate predictors based on data collected in the first 6 or 12 months post-transplantation, separately. Specifically, eGFR was calculated using the CKD-EPI equation, which is based on serum creatinine (Scr, μmol/L), gender, and age [34]. NLR, PLR, and MLR were generated via the divisions of neutrophil count, platelet count, and monocyte count by lymphocyte count, respectively. Average values of red blood cell count (RBC), hemoglobin (HGB), hematocrit (HCT), platelet count (PLT), white blood cell count (WBC), neutrophil percentage and absolute number, lymphocyte percentage and absolute number, monocyte percentage and absolute number, NLR, PLR, MLR, uric acid level and TAC concentration were calculated using all data measured within 0–6 or 0–12 months post-transplant. The coefficient of variations (CV) of TAC and eGFR were generated to reflect the relative variability of TAC and eGFR levels within a certain period of time [CV (%) = (Standard Deviation/Mean) * 100]. In addition, we calculated the number of times the TAC trough concentration fell within, below, or beyond the target range of 5–10 ng/ml in the first 3 months and 4–8 ng/ml during months 4–12. The percentage of each condition relative to total TAC test times were recorded as TAC-TTR, TAC-loLL and TAC-hiUL. Considering the rapid recovery of renal function after KT surgery and the large fluctuation that inherently exists in all KTRs during the first month, we calculated eGFR-Mean and eGFR-CV by using data from 1 to 6 or 12 months post-transplantation to maximally reflect their actual predictive potential. For routine urine tests, we retrieved semi-quantitative results of urine protein at months 6 and 12 post-transplant.

The CBC tests were completed with Sysmex XN9100 automated hematology system (Sysmex Corporation, Japan). Urine proteins were semi-quantified with fully automated urine chemistry analyzer UF5000 plus UC3500 (Sysmex Corporation, Japan). Scr and uric acid levels were determined with cobas 8000 modular analyzer (Roche Company, Switzerland). TAC trough concentration was assessed with V-TWIN analyzer system (Siemens, Germany).

Patients with missing one-year follow-up laboratory data were excluded at the enrollment stage. A multiple imputation method was utilized to impute suitable values for missing data in demographic and clinical variables.

### Predictor selection, model development, and validation

In the training cohort, predictors were selected into final models through three steps (Figure 1). First, we conducted univariate Cox proportional hazards regression analysis to determine associations between candidate predictors and 5-year graft loss. Then we picked out factors with *P* < 0.15 and stratified them as binary variables based on optimal cut-off values obtained from X-tile analysis [35] (Supplementary Figure 1). Two model feature selections were implemented in parallel (model 1: demographics and 0–6m laboratory variables; model 2: demographics and 0–12m laboratory variables). Least absolute shrinkage and selection operator (LASSO) regression analysis was further performed to select valuable predictors among the above-stratified variables. LASSO performs L1 regulation, which adds a penalty to the absolute value of the magnitude of coefficients. As a tuning parameter, λ controls the strength of the L1 penalty. We kept variables with non-zero coefficients for the following analysis by setting λ equal to the minimum value, for which the cross-validation error was the smallest. Finally, multiple Cox hazards regression analysis (forward LR) was added to determine final predictors in individual models and forest plots were drawn to demonstrate the estimated hazard ratio (HR) of each variable.

Models that incorporated the selected independent predictors were visually presented as nomograms. Harrell’s concordance index (C-index) and the dynamic time-dependent area under the receiver operating characteristic curves (TD-AUC) were used to evaluate the discrimination power of nomograms in training and validation cohorts. The higher the C-index and TD-AUC values, the better the predictive accuracy. Calibration ability was assessed by graphically plotting the actual probabilities and nomogram-predicted probabilities via a bootstrapping method with 1000-iteration resampling. In addition, decision curve analysis (DCA) was utilized to evaluate the clinical utility of nomograms by calculating the net benefit of a range of threshold probabilities in training and validation cohorts [36].

### Risk classification of KTRs

In the training cohort, total risk points of individual patients were calculated based on linear predictor values and the point per unit of linear predictor, which were directly available in the nomogram scoring system. We applied X-tile software (version 3.6.1, Yale University, New Haven, CT, USA) to determine optimal cut-off values of the risk point, with which patients were categorized into low-, moderate-, and high-risk groups. The Kaplan-Meier curves were performed to present the 5-year graft survival of different risk groups. Differences between groups were statistically compared with the aid of the log-rank test.

### Statistical analysis

Data was demonstrated as absolute number, mean ± standard deviation, or median (interquartile range) according to type. Chi-square or Fisher’s exact tests were utilized to compare categorical variables between groups. The student's *t*-test and Mann-Whitney *U* test were applied to compare continuous variables with normal distribution and skewed distribution, respectively. All statistical analyses were completed with SPSS software (version 23.0, SPSS Inc., Chicago, IL, USA) and R software (version 3.6.3, https://cran.r-project.org). R packages “glmnet”, “rms”, “survivalROC”, “ggplot2”, “stdca.R”, “survminer”, “DynNom” and “shiny” were used in this study. A two-tailed *P* < 0.05 was considered statistically significant. All analyses were reported according to the Transparent Reporting of a multivariate prediction model for Individual Prognosis Or Diagnosis (TRIPOD) statement [37].

## Supplementary Material

Supplementary Figures
