# Supplementary material for: Development and validation of routine clinical laboratory data derived marker-based nomograms for the prediction of 5-year graft survival in kidney transplant recipients
Source: Aging (Albany NY). 2021 Mar 26;13(7):9927–47. doi: 10.18632/aging.202748 (PMC8064213; doi:10.18632/aging.202748)
Supplement: Supplementary Figures [file aging-13-202748-s001.pdf]

SUPPLEMENTARY FIGURES

1. Clinical variables

A Age (years)

Optimal cutoff value = 35

HR (< 35)=2.27

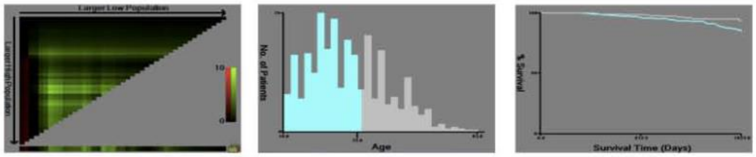

B Hospitalization time (days)

Optimal cutoff value = 34

HR (>34)=1.47

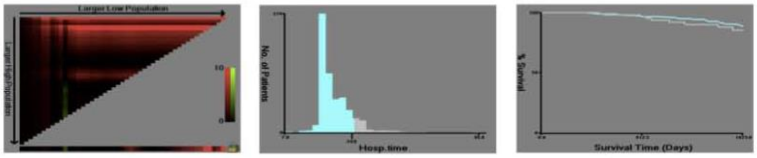

C BMI (kg/m^2)

Optimal cutoff value = 25.3

HR (>25.3)=2.63

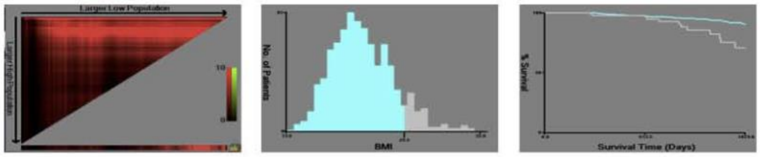

2. Laboratory indexes within 6 months after KT

A TAC-TTR (%)

Optimal cutoff value = 58.3

HR (<58.3)=1.98

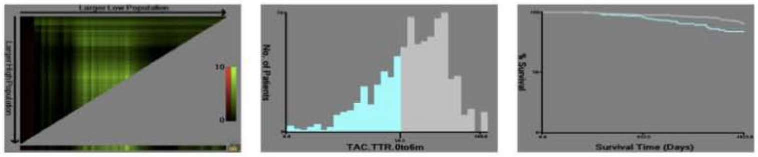

B eGFR-Mean (mL/min/1.73m^2)

Optimal cutoff value = 58.4

HR (<58.4)=2.58

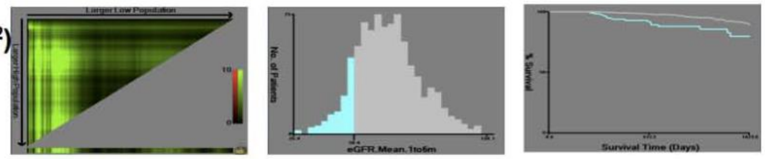

C eGFR-CV (%)

Optimal cutoff value = 13.7

HR (>13.7)=2.45

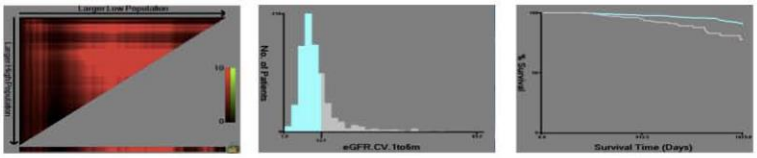

D Uric acid-Mean (umol/L)

Optimal cutoff value =357.8

HR (>357.8)=1.8

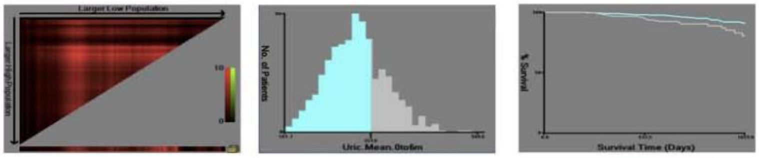

E WBC-Mean (10^9/L)

Optimal cutoff value = 5.9

HR (>5.9)=3.09

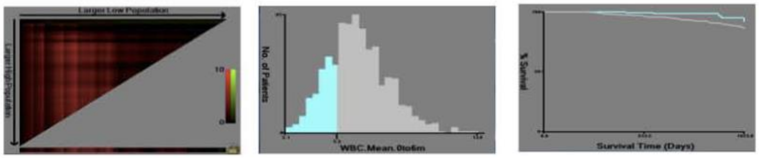

F Lymphocyte-Mean (%)
Optimal cutoff value =29.7
HR (<29.7)=2.77

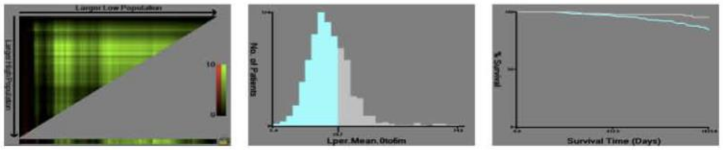

G Neutrophil-Mean (%)
Optimal cutoff value = 60.8
HR (>60.8)=3.48

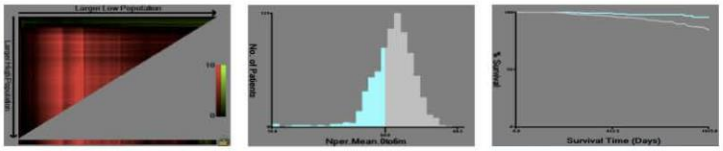

H Neutrophil-Mean (10^9/L)
Optimal cutoff value = 4.7
HR (>4.7)=2.64

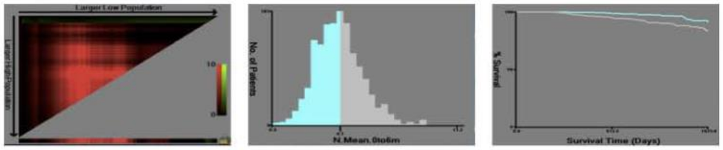

I NLR-Mean
Optimal cutoff value = 2.7
HR (>2.7)=2.4

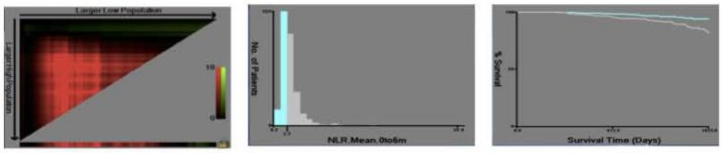

3. Laboratory indexes within 12 months after KT

A TAC-CV (%)
Optimal cutoff value = 49.8
HR (>49.8)=2.53

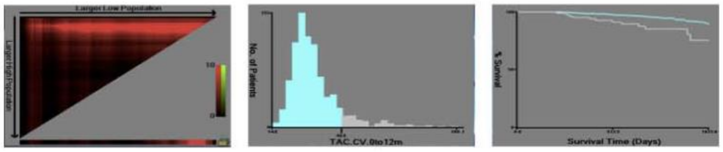

B TAC-TTR (%)
Optimal cutoff value = 59.6
HR (<59.6)=1.98

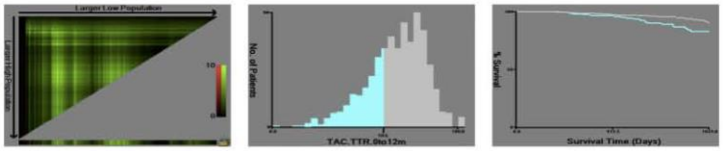

C TAC-loLL (%)
Optimal cutoff value = 48.5
HR (>48.5)=2.24

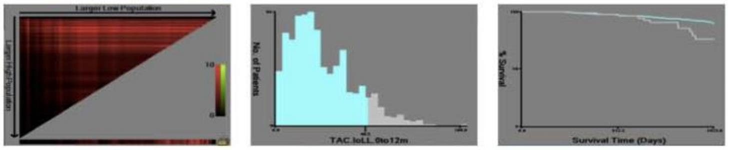

D eGFR-Mean (mL/min/1.73m^2)
Optimal cutoff value = 59.1
HR (<59.1)=4.68

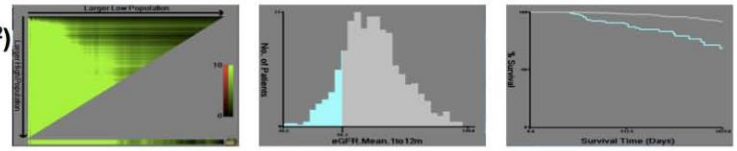

E eGFR-CV (%)
Optimal cutoff value = 16.6
HR (>16.6)=5.03

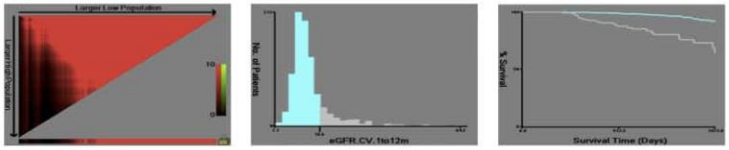

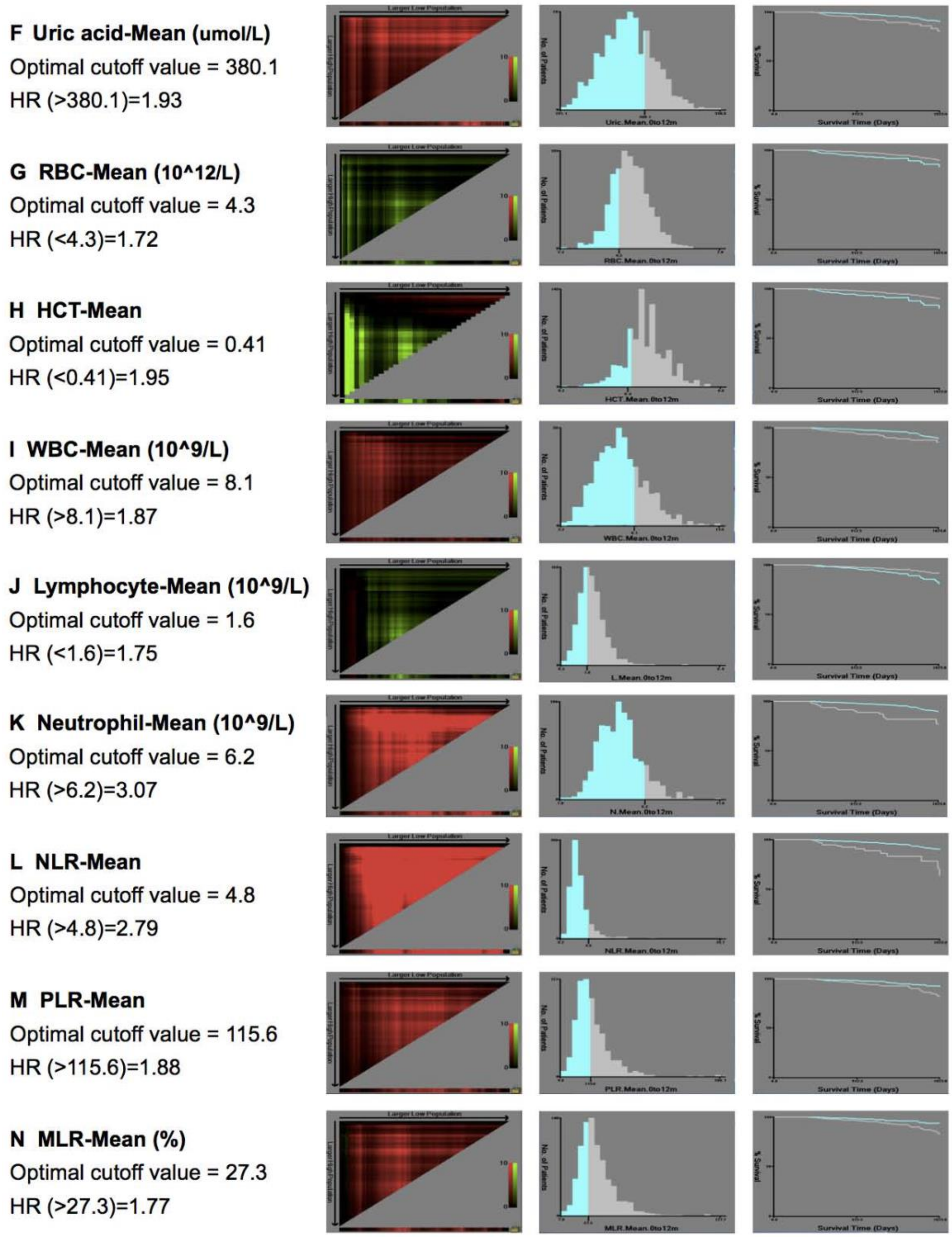

Supplementary Figure 1. Determination of optimal cut-off values for clinical and laboratory variables whose *P* values less than 0.15 in univariate Cox analysis.

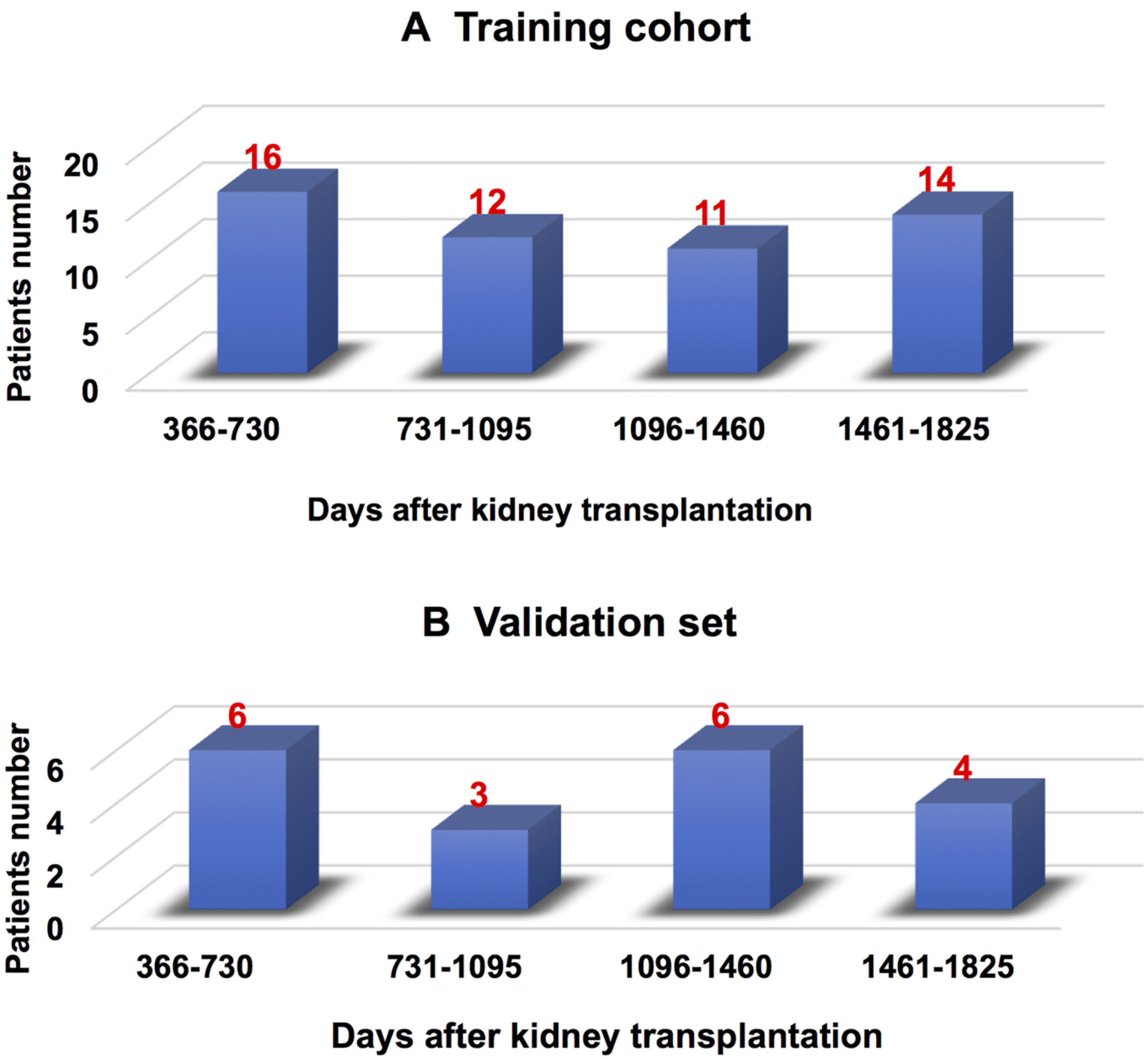

Supplementary Figure 2. Time point distribution of occurring graft loss in training (**A**) and validation (**B**) cohorts.

Laboratory indexes calculators for predicting 5-year graft survival in kidney transplant recipients (Results region)				
**BMI**	**TAC-CV**	**eGFR-CV**	**Neutrophil (%)-Mean**	**Lymphocyte absolute numer (10^9/L)-Mean**
**BMI (kg/m2)**	**TAC-CV (%)**	**eGFR-CV (%)**	**Neutrophil (%)-Mean**	**Lymphocyte (abs)-Mean**
**19.53125**	**20.24440825**	**11.86930454**	**68.06666667**	**0.8666666667**
	**TAC-Mean (ng/mL)**	**eGFR-Mean (mL/min/1.73m2)**	**Neutrophil (%)-times**	**Lymphocyte (abs)-times**
	6.1	108.3908428	3	3
	**TAC-SD**	**eGFR-SD**		
	1.234908904	12.86523923		
	**TAC-times**	**eGFR-times**		
	5	5		

Note: enter your original laboratory values into pink boxes (Input region)					
**Height (cm)**	**TAC C0 values (ng/mL)**	**Gender (M/F)**	**Age (years)**	**Neutrophil (%) values**	**Lymphocyte (abs) values (10^9/L)**
160	5.7	M	30	73.6	0.64
**Weight (kg)**	4.6	**Scr values (μmol/L)**	**eGFR levels (mL/min/1**	63.1	0.81
50	5.9	78	117.8579954	67.5	1.15
	6.3	79	117.2425342		
	8	100	89.05857488		
		80	116.6379685		
		90	101.1571412		
			#NUM!		
			#NUM!		
			#NUM!		
			#NUM!		
			#NUM!		
			#NUM!		
			#NUM!		
			#NUM!		
			#NUM!		

Supplementary Figure 3. Interface example of laboratory indexes calculator for the calculation of predictors for predicting 5-year graft survival in KTRs. Please visit website https://docs.google.com/spreadsheets/d/1IJX9YZBTON1xwVrNYp5PzcNpWQ1CItGm-N_nDOKxbpk/edit?pli=1#gid=0.
